# Exploring Parental Experiences With School-Aged Children Receiving Web-Based Learning: Cross-Sectional Study

**DOI:** 10.2196/50892

**Published:** 2023-12-19

**Authors:** Samaa Al Anazi, Eman Bajamal, Neama Hantira, Ola Esheaba

**Affiliations:** 1 College of Nursing, King Saud Bin Abdulaziz University for Health Sciences, Jeddah, Saudi Arabia; 2 King Abdullah International Medical Research Center, Jeddah, Saudi Arabia; 3 Faculty of Nursing, Alexandria University, Alexandria, Egypt

**Keywords:** education, obstacles, online learning, stress reduction, obstacle, parent, parents, parental, parenting, e-learning, child, children, school, schools, student, students, experiences, experience, interaction, health outcome, health outcomes, family, dynamic, dynamics, parental experiences

## Abstract

**Background:**

Web-based learning has transformed education. Its ability to overcome physical barriers and deliver knowledge at the click of a button has made web-based learning popular and ensured that it will continue to be used in the future. The involvement of parents in web-based learning is fundamental to the success of the educational process, but limited attention has been paid to the impact of web-based learning on parents.

**Objective:**

This study examined parental experiences with school-aged children receiving web-based learning in Jeddah, Saudi Arabia.

**Methods:**

We sent cross-sectional, anonymous web-based questionnaires to school-aged children’s parents. A total of 184 parents completed the survey.

**Results:**

Parents’ negative experiences of web-based learning (mean 4.13, SD 0.62) exceeded their positive experiences (mean 3.52, SD 0.65). The most negative experience reported by parents was their child’s boredom due to prolonged sitting in front of a device (mean 4.56, SD 0.69). The most positive experience was their child’s technological skill enhancement (mean 3.98, SD 88). Their child’s lack of social interaction and friendship building promoted stress among parents (*r*=−0.190; *P*=.01). At the same time, their child’s technological skill enhancement reduced stress among parents (*r*=0.261; *P*=.001). The most reported (63/184, 34.2%) obstacle to web-based learning was having multiple learners in the same household.

**Conclusion:**

Web-based learning is a fundamental learning method and will continue to be used in the future because of its ability to overcome many barriers to education. Parental involvement in the continuity and success of the web-based learning process is crucial. However, the findings of this study illustrated that parents’ experiences of web-based learning were more negative than positive. Parents who reported negative experiences reported an increase in stress and faced more obstacles due to web-based learning. Thus, more attention and intervention are needed to promote positive web-based learning experiences among parents.

## Introduction

In December 2019, a new strain of the coronavirus emerged [1], and many countries enforced anticontagion policies to slow the virus’s spread [2]. Over 107 countries around the world closed their schools, causing 862 million children and young adults to remain home during the pandemic [3].

The Ministry of Education in the Kingdom of Saudi Arabia (KSA) suspended traditional face-to-face classes and implemented web-based learning to enforce social distancing policies; ensure the safety of teachers, students, and the community; and maintain the continuity of learning [4]. Web-based learning in the KSA included live sessions with student or recorded classes. It relied heavily on the provision of internet access to all students and teachers [5]. Additionally, the Ministry of Education used TV to broadcast lessons to students who lacked internet access. Over 127 teachers broadcast lessons to 19 TV channels [6]. The digitalization of education in the KSA during COVID-19 was essential to maintain students’ academic achievements. It included the development of over 19 applications to serve the public, including the education sector. Collaboration between the government and telecommunication companies was initiated [6].

The shift in the educational process forced parents and children to transform their way of life, creating many advantages and disadvantages [7]. Web-based learning was introduced across the world, including in India [5], Pakistan [8], Indonesia [9], New Zealand [10], and Jordan [11]. It placed parents in an unusual situation, requiring increased parental involvement in children’s education, compensation of children’s social and interactional needs, and rearrangement of priorities to ensure the continuity of academic achievements [5,8].

Because of the gravity of COVID-19 and its devastating consequences, many parents agreed to the KSA’s school closure and web-based learning policy. However, the parents faced multiple obstacles to web-based learning, such as an inability to manage several responsibilities, an inability to motivate children to carry out web-based learning, and feelings of uncertainty about web-based learning’s effectiveness [12].

Parents in Indonesia reported the inability of their children to focus on their classes. The children felt bored, and parents were not able to motivate them [13]. Further, parents could not enforce web-based learning because children did not take it seriously, and they had limited understanding of the educational material [13].

Multiple factors affect parental involvement and web-based learning management, including the lack of communication between parents and teachers, lack of time to manage teaching and homework, and technological difficulties [14]. Parents also lack teaching skills and have little knowledge of educational materials [14].

Parents can use adaptation strategies for web-based learning. Coping strategies can include the establishment of a daily routine, an increase in physical activities, and positive reappraisal [15]. In studies of mindfulness and cognitive therapy, stress reduction is defined as the individual’s ability to accept any unpleasant situation and promote a relationship between themselves and stressful ideas through emotional regulation [16].

As time passed and pandemic restrictions were lifted, the global perception toward educational systems changed. Many schools and universities started to view web-based learning as a new and important approach to overcome physical distance as a barrier to education.

**Figure 1.**
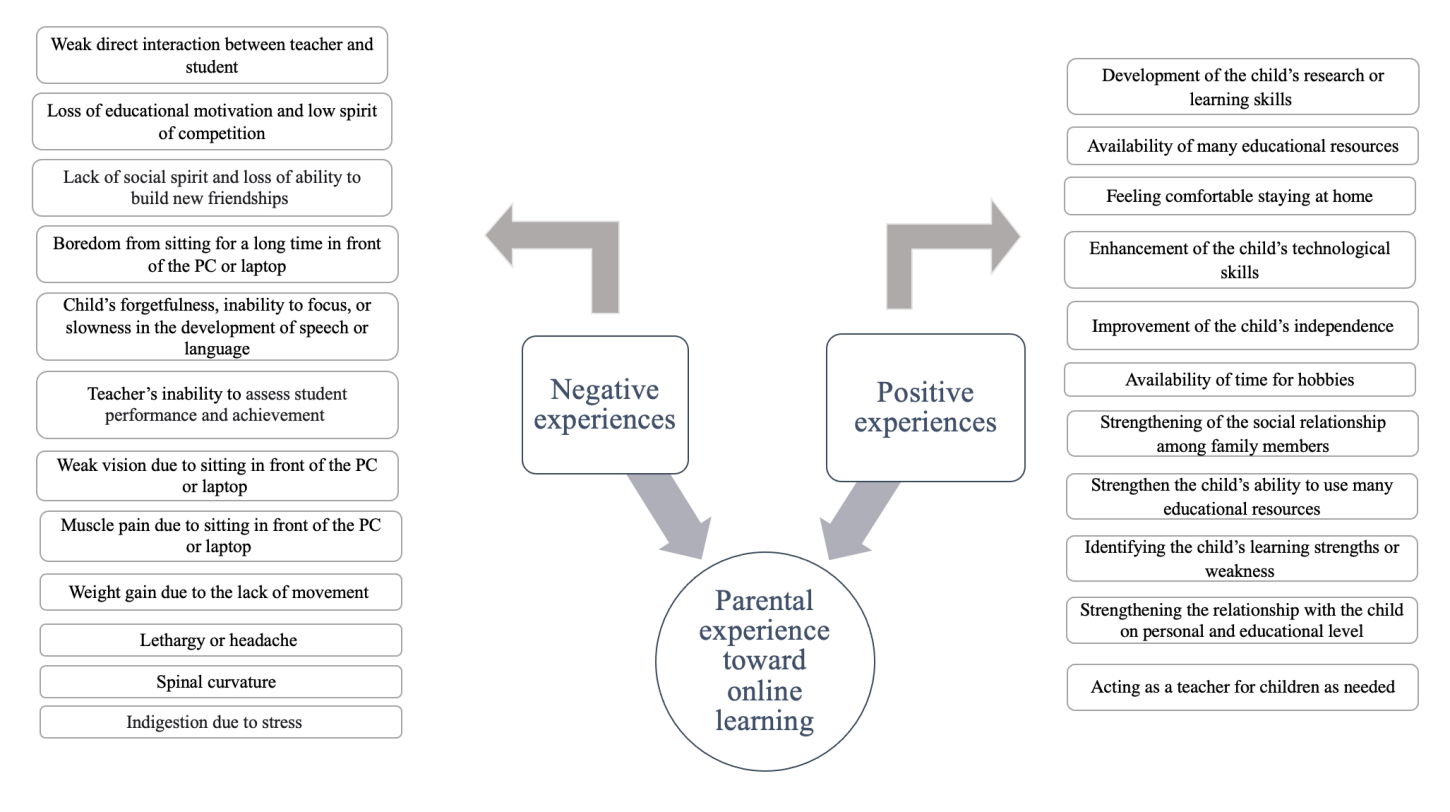
Parental experiences of web-based learning.

Literature on the impact of web-based learning on parents and their children is still lacking in Saudi Arabia. Parental experiences of the shift in their children’s educational processes from traditional classes to web-based learning need further investigation. This study aimed to explore parents’ self-identified positive and negative experiences of web-based learning as a teaching method for their school-aged children. Figure 1, adapted from Garbe et al [12], shows the positive and negative experiences parents face during web-based learning [12]. We examined these experiences and their impact on stress reduction after 1 year of web-based learning and reported the obstacles they found. The results of this study will guide future research on the impact of web-based learning on parents and will help stakeholders formulate policies and procedures to enhance web-based learning and overcome identified obstacles.

## Methods

### Design

A quantitative cross-sectional study design was used to explore parents’ positive and negative experiences of web-based learning and parents’ stress after 1 year of web-based learning. The relationship between the learning obstacles that parents reported and parents’ negative web-based learning experiences was also examined.

### Instrument

Data were collected through a web-based survey. The survey and informed consent form were sent electronically to participants after obtaining institutional review board (IRB) approval from King Abdullah International Medical Research Center. The survey consisted of questions about parents’ demographics (10 questions), their school-aged children (13 questions), their overall positive and negative web-based learning experiences (13 items for negative experiences and 11 items for positive experiences), their stress reduction regarding web-based learning (3 questions), and the obstacles to web-based learning that they observed (3 questions).

Every item in the survey was graded on a 5-point Likert scale with 5 responses: strongly disagree (score of 1 point), disagree (2 points), neutral (3 points), agree (4 points), and strongly agree (5 points). Higher scores were associated with increased perception of a variable. The survey was adapted from Garbe et al [12] and then modified and translated to Arabic. Bajamal et al [17] used the Arabic version to examine parents’ positive or negative experiences of web-based learning. Internal consistency via Cronbach α was reported at .890 and .892, with a confirmed correlation coefficient ranging from 0.52 to 0.73 and from 0.43 to 0.76. Additionally, exploratory factor analysis demonstrated a single factor with a total percentage variance of 52.89 for positive experiences and 56.83 for negative experiences [17].

### Sample, Setting, and Data Collection

Participants were recruited using nonprobability snowball sampling. The study targeted parents with school-aged children who were enrolled in web-based classes during the COVID-19 pandemic in Jeddah, Saudi Arabia.

Those who met all of the following criteria were eligible to participate in the study: (1) mother or father of school-aged children taking web-based courses, (2) resident of Jeddah city, (3) ability to speak and read Arabic, and (4) ≥18 years of age. Participants filled out the informed consent form prior to answering the survey. The exclusion criteria were not being a parent, inability to speak and read Arabic, and younger than 18 years of age. Potential participants were recruited via snowball sampling on WhatsApp and Facebook. After the potential participants filled out the survey, the researchers identified those who met the inclusion criteria and enrolled them in the study. If participants agreed to participate in the survey after reading the informed consent form, they were directed to the survey page directly. For this descriptive study, the α significant level was set at .05 with a power of 0.8. The sample size was estimated using G*Power software (Heinrich-Heine-Universität Düsseldorf). This study was the first to examine Saudi parents’ experiences with web-based learning and the effect of obstacles to web-based learning on these experiences during the COVID-19 pandemic. No effect size was used when conducting the power analysis. In cases such as this study, where an effect size has not been reported in the literature, the estimation of effect size is based on the researcher’s logic and judgment [18]. Therefore, a medium effect size of 0.05, power of 0.80, and α significance level of .05 were used to guide the statistical analysis. The targeted sample size was set at 180 parents, with the percentage of missing data presumed to be 10%. The total targeted sample size was as follows: N = 180 + 18 = 198.

### Data Analysis

SPSS computer software (SPSS for Mac, version 28.0; IBM Corp) was used to analyze the data. Different types of analysis were proposed for this study. Descriptive statistics were calculated for all variables of interest, including means, SDs, frequencies, and percentages. In regard to inferential statistics, Pearson correlation coefficients were used to examine the relationships among continuous variables. Spearman correlation was used to examine the relationship among the survey items. Statistical significance was based on the standard α level of .05.

### Ethical Considerations

This study received ethics approval from the IRB of the King Abdullah International Medical Research Center (NRJ21J/111/04).

Electronic informed consent followed the same template of the informed consent form filed to the IRB committee. Electronic signature occurred via checking a box next to the following statement: “By checking this box, I hereby sign this informed consent electronically.” Researchers’ contact information was available in case questions were raised regarding the study. Potential participants were notified about the voluntary nature of participation in the study and its descriptive purpose. The fact that participation in the study involves minimum risks was also added to the consent form.

To ensure confidentiality, electronic data are stored in a computer protected by 2 passwords. Researchers used the collected data for the purpose of the study only, and data access was granted to study personnel only. Participants’ data were anonymous. No compensation or incentive was delivered to the participants.

## Results

### Participant Characteristics

As Table 1 shows, a total of 184 participants completed the survey. Of these participants, 139 (75.5%) were mothers. Most (n=98, 53.3%) of the participants were in the age group of 30-40 years. More than half (n=110, 59.8%) of the participants had an undergraduate degree.

**Table 1.** The sample’s demographic characteristics (N=184). There were missing data (n=83, 45.1%) for working hours, work shifts, and occupation related to education.

Demographic variable		Participant, n (%)	
**Person completing the survey**			
	Mother	139 (75.5)	
	Father	40 (21.8)	
	Nonparent	5 (2.7)	
**Age group (y) **			
	<30	7 (3.8)	
	30-40	98 (53.3)	
	40-50	64 (34.8)	
	>50	15 (8.2)	
**Educational level**			
	Primary school	5 (2.7)	
	Intermediate school	2 (1.1)	
	High school	23 (12.5)	
	Undergraduate degree	110 (59.8)	
	Postgraduate degree	44 (23.9)	
**Family income (SR $; SR $1=US 0.26$)**			
	<3000	8 (4.3)	
	3000-5000	10 (5.4)	
	5000-8000	25 (13.6)	
	8000-12,000	33 (18)	
	>12,000	108 (58.7)	
**Residency status**			
	Family house	48 (26.1)	
	Separate apartment	136 (73.9)	
**Person following web-based learning**			
	Mother only	121 (65.8)	
	Father only	4 (2.2)	
	Mother and father together	53 (28.8)	
	Another family member	6 (3.3)	
**Parent occupation status**			
	Working	101 (54.9)	
	Not working	83 (45.1)	
**Working hours (n=101)**			
	Full-time	86 (85.1)	
	Part-time	15 (14.9)	
**Work shift (n=101)**			
	Day	91 (90.1)	
	Night	10 (9.9)	
**Occupation related to education (n=101)**			
	Yes	49 (48.5)	
	No	52 (51.5)	
**Parents deal with technology**			
	Yes	164 (89.1)	
	Somewhat	20 (10.9)	
**Internet connection during school closure**			
	Yes	180 (97.8)	
	No	4 (2.2)	
**Number of children**			
	1	15 (8.2)	
	2	48 (26.1)	
	3	51 (27.7)	
	>3	70 (38)	
**Number of school-aged children**			
	1	35 (19)	
	2	64 (34.8)	
	3	53 (28.8)	
	>3	15 (8.2)	
	All^a^	17 (9.2)	

a All children in the household, regardless of the total number of children, are school-aged and underwent web-based learning.

Further, more than half (n=136, 73.9%) of the participants lived in a separate home or apartment. Most (n=121, 65.8%) of the participants who followed up with their children for web-based learning were mothers.

Moreover, most (n=101, 54.9%) of the participants who followed up with their children for web-based learning were currently working. Most (91/101, 90.1%) of them were working during the day. More than half (52/101, 51.5%) of the participants’ jobs were not related to the education field.

During school closure, almost all participants (180/184, 97.8%) had an internet connection. Almost all participants (n=164, 89.1%) knew how to use web-based learning technology.

As Table 2 shows, most (77/184, 41.8%) of the children who needed follow-ups by their parents during web-based learning were enrolled in elementary school, followed by children from elementary or intermediate school (n=33, 17.9%) and children from elementary, intermediate, or high school (n=24, 13.0%). Most (n=158, 85.9%) of these children shifted to web-based classes during COVID-19; only 26 (14.1%) participants reported that their children went to hybrid classes. During web-based learning, all children needed support from their parents. Most children (n=56, 30.4%) needed a total of 60-120 minutes of support, and the smallest proportion of children (n=34, 18.5%) needed 120-180 minutes of support.

**Table 2.** Academic profile of the children (N=184).

Demographic variable		Children, n (%)	
**Education level**			
	Kindergarten	6 (3.3)	
	Elementary school	77 (41.8)	
	Intermediate school	12 (6.5)	
	High school	5 (2.7)	
	Elementary or intermediate school	33 (17.9)	
	Elementary or high school	16 (8.7)	
	Intermediate or high school	4 (2.2)	
	Elementary, intermediate, or high school	24 (13)	
	University level	7 (3.8)	
**Fully dependent during web-based classes**			
	Yes	158 (85.9)	
	Hybrid	26 (14.1)	
**Support time from parents (min)**			
	<60	44 (23.9)	
	60-120	56 (30.4)	
	120-180	34 (18.5)	
	>180	50 (27.2)	

As Table 3 shows, during school closure, participants spent more time with their children doing activities such as school activities (117/184, 63.6%), web-based activities (n=67, 36.4%), watching TV (n=66, 35.9%), and nonschool activities (n=45, 24.5%).

**Table 3.** Activities parents did with their children (N=184).

Activities	Participant, n (%)	
School activities	117 (63.6)	
Web-based activities	67 (36.4)	
TV	66 (35.9)	
Nonschool activities	45 (24.5)	
Reading	33 (17.9)	
Outdoor activities	29 (15.8)	
Other activities	27 (14.7)	
Indoor games	26 (14.1)	

Table 4 shows which courses parents felt should be given priority. Table 5 shows parents’ feelings and experiences of school closure (mean 3.88, SD 1.13) and toward school support during school closure (mean 3.39, SD 1.03).

**Table 4.** Subjects that parents felt should be prioritized during school closure (N=184).

Subjects	Participant, n (%)	
Reading	142 (77.2)	
Mathematics	135 (73.4)	
Writing	120 (65.2)	
Sciences	85 (46.2)	
Others	86 (46.8)	

**Table 5.** Descriptive statistics for parents’ feelings.

Parents’ feelings	Score, mean (SD)	
Parents’ feelings about school closure	3.88 (1.13)	
Parents’ feelings about school support	3.39 (1.03)	

Table 6 shows the most reported obstacles parents observed with regard to web-based learning. The highest reported obstacle was having multiple web-based learners in the same household at the same time (63/184, 34.2%). This was followed by time constraints (n=58, 31.5%) and having an uncooperative child (n=47, 25.5%).

**Table 6.** Reported obstacles (N=184).

Obstacles	Participant, n (%)	
Time constraints	58 (31.5)	
Financial concerns	16 (8.7)	
Noncooperative child	47 (25.5)	
Many web-based learners in the same household	63 (34.2)	

### Descriptive Statistics of Negative and Positive Experiences

The below data illustrate the overall positive and negative experiences of parents. The mean of negative experiences of web-based learning exceeded that of positive experiences, indicating that parents required more interventions to better equip them to manage web-based learning.

As shown in Table 7, the total score of the negative experiences of parents regarding web-based learning was mean 4.13 (SD 0.62). The highest mean score was reported for feeling bored after sitting for a long time in front of a PC or laptop at 4.59 (SD 0.69). This was followed by a lack of social spirit and the loss of the ability to build new friendships at 4.49 (SD 0.75). The lowest mean score was reported for indigestion due to stress at 3.43 (SD 1.05), indicating that parents’ negative experiences of web-based learning did not include physiological symptoms.

**Table 7.** Descriptive statistics for negative experiences of web-based learning (N=184).

Negative experiences	Score, mean (SD)	
Weak direct interaction between teacher and student	4.24 (0.87)	
Loss of educational motivation and low spirit of competition	4.25 (0.90)	
Lack of social spirit and loss of ability to build new friendships	4.49 (0.75)	
Boredom from sitting for a long time in front of the PC or laptop	4.59 (0.69)	
Child’s forgetfulness, inability to focus, or slowness in the development of speech or language	4.21 (0.91)	
Teacher’s inability to assess student performance and achievement	3.91 (1.01)	
Weak vision due to sitting in front of the PC or laptop	4.19 (0.92)	
Muscle pain due to sitting in front of the PC or laptop	4.16 (0.92)	
Weight gain due to the lack of movement	4.05 (1.03)	
Lethargy or headache	4.04 (0.95)	
Spinal curvature	3.97 (1.00)	
Indigestion due to stress	3.43 (1.05)	
Total negative experience	4.13 (0.62)	

Next, as Table 8 shows, the total score for the positive experiences of parents regarding web-based learning (mean 3.52, SD 0.65) was lower than that for negative experiences (mean 4.13, SD 0.62). Out of all the positive experiences of web-based learning, the enhancement of the child’s technological skills had the highest reported mean score at 3.98 (SD 0.88). The lowest reported mean score was 3.05 (SD 0.95) for available time for hobbies.

**Table 8.** Descriptive statistics for positive experiences of web-based learning (N=184).

Positive experiences	Score, mean (SD)	
Development of the child’s research or learning skills	3.59 (0.92)	
Availability of many educational resources	3.50 (0.88)	
Feeling comfortable staying at home	3.54 (1.04)	
Enhancement of the child’s technological skills	3.98 (0.88)	
Improvement of the child’s independence	3.60 (1.07)	
Availability of time for hobbies	3.05 (0.95)	
Strengthening of the social relationship among family members	3.38 (0.97)	
Strengthening of the child’s ability to use many educational resources	3.63 (0.91)	
Identifying the child’s learning strengths or weaknesses	3.50 (0.98)	
Strengthening the relationship with the child on a personal and educational level	3.46 (0.95)	
Acting as a teacher for children as needed	3.50 (0.88)	
Total positive experiences	3.52 (0.65)	

### Association Between Negative or Positive Experiences, Stress Reduction, and Obstacles to Web-Based Learning

Table 9 shows the Pearson product-moment correlations among the study variables. Weak correlation was found between the variables of stress reduction after 1 year of web-based learning and the experiences of parents. However, stress reduction after 1 year of web-based learning was positively correlated with parents’ positive experiences (*r*=0.237; *P*<.01) more often than with parents’ negative experiences. Further, obstacles reported by parents positively correlated with their negative experiences (*r*=0.209; *P*=<.01). The results showed that parents’ experiences of web-based learning affected their stress reduction and the obstacles they reported.

**Table 9.** Pearson product-moment correlations among positive experiences, negative experiences, stress reduction, and obstacles toward web-based learning (N=184).

Variables				Stress reduction after 1 year	Negative experience	Positive experience	Obstacles
**Stress reduction after 1 year**							
	*r*			1	–0.121	0.237	0.017
	*P* value			—^a^	.10	.001	.82
**Negative experience**							
	*r*			–0.121	1	–0.378	0.209
	*P* value			.10	—	<.001	.004
**Positive experience**							
	*r*			0.237	–0.378	1	–0.101
	*P* value			.001	<.001	—	.17
**Obstacles**							
	*r*			0.017	0.209	–0.101	1
	*P* value			.82	.004	.17	—

a Not applicable.

### Association Between Negative Experiences and Stress Reduction

Table 10 shows the Spearman correlations between negative experiences and stress reduction after 1 year of web-based learning. The results showed poor correlation between stress reduction and negative experiences.

**Table 10.** Spearman correlations between negative experiences and stress reduction 1 year after web-based learning (N=184).

Variable	Stress reduction after 1 year, *r*	*P* value
Stress reduction after 1 year	1.00	N/A^a^
Weak direct interaction between teacher and student	−0.114	.12
Loss of educational motivation and low spirit of competition	−0.043	.56
Lack of social spirit and loss of ability to build new friendships	−0.190	.01
Boredom from sitting for a long time in front of the PC or laptop	−0.129	.08
Child’s forgetfulness, inability to focus, or slowness in the development of speech or language	−0.050	.50
Teacher’s inability to assess student performance and achievement	−0.018	.81
Weak vision due to sitting in front of the PC or laptop	−0.080	.28
Muscle pain due to sitting in front of the PC or laptop	−0.035	.64
Weight gain due to the lack of movement	0.007	.93
Lethargy or headache	−0.056	.45
Spinal curvature	−0.191	.009
Indigestion due to stress	−0.170	.02
Negative experience score	−0.161	.03

a N/A: not applicable.

According to Table 10, stress reduction after 1 year of web-based learning was negatively correlated with spinal curvature (*r*=−0.191; *P*=.009) and the lack of social spirit and loss of ability to build new friendships (*r*=−0.190; *P*=.01), followed by indigestion due to stress (*r*=−0.170; *P*=.02) and the total negative experience score (*r*=−0.161; *P*=.03).

### Association Between Positive Experiences and Stress Reduction

Table 11 shows the Spearman correlations among the positive experience items. Stress reduction after 1 year of web-based learning was positively correlated with the enhancement of the child’s technological skills (*r*=0.261; *P*<.001), followed by strengthening of the child’s ability to use many educational resources (*r*=0.219; *P*=.003), improvement of the child’s independence (*r*=0.172; *P*=.02), strengthening of the social relationship between family members (*r*=0.171; *P*=.02), development of the child’s research or learning skills (*r*=0.157; *P*=.03), and the total positive experience score (*r*=0.194; *P*=.008).

**Table 11.** Spearman correlations between positive experiences and stress reduction 1 year after web-based learning (N=184).

Variable	Stress reduction after 1 year, *r*	*P* value
Stress reduction after 1 year	1.00	N/A^a^
Development of the child’s research or learning skills	0.157	.03
Availability of many educational resources	0.131	.08
Feeling comfortable staying at home	0.141	.06
Enhancement of the child’s technological skills	0.261	<.001
Improvement of the child’s independence	0.172	.02
Availability of time for hobbies	0.064	.39
Strengthening of the social relationship among family members	0.171	.02
Strengthening of the child’s ability to use many educational resources	0.219	.003
Identifying the child’s learning strengths or weaknesses	−0.003	.96
Strengthening the relationship with the child on a personal and educational level	0.129	.08
Acting as teacher for children as needed	0.115	.12
Positive experience score	0.194	.008

a N/A: not applicable.

## Discussion

### Principal Findings

This study was the first to examine parents’ reported experiences of their children’s web-based learning in the city of Jeddah, Saudi Arabia. Parents’ reported obstacles to web-based learning and stress reduction after 1 year of web-based learning were also explored. Our results indicated that parents’ negative experiences exceeded their positive ones. Additionally, parents who had positive experiences reported less stress after 1 year of web-based learning. Parents with negative experiences reported more obstacles to web-based learning compared to parents with positive experiences.

Web-based learning increased parents’ responsibilities and their crucial role in web-based learning, causing them to face many challenges and difficulties. Findings from 2 international studies conducted in China and Indonesia examined parental experiences of web-based learning during the COVID-19 pandemic. Their findings agreed with this study’s findings [19,20].

Out of all the negative experiences reported by parents, children’s boredom from sitting in front of electronic devices scored the highest. Children’s prolonged use of electronic devices to attend classes and do their homework led to a sense of boredom and decreased motivation. This sense of boredom can pose a great challenge to parents who aim to increase their child’s interest and maintain their curiosity about the learning process. In 2 previous studies in Indonesia, parents used the same term, “boredom,” to describe their child’s feelings during web-based learning. Child boredom is associated with web-based learning and with the quarantine during the COVID-19 lockdown [21,22].

Parents recognized that the global pandemic led to the transformation of education and that with every hardship their child encountered, they had a positive experience or gained a skill. Skills such as independence in the learning process and the use of multiple educational resources remained crucial after the pandemic ended because web-based learning did not stop. Parents’ positive attitudes and perception toward web-based learning has been proven in studies conducted in India and Indonesia [23,24]. In both countries, parents reported enhanced parental engagement in their children’s education and noted the ability of web-based learning to allow education to persist despite social distancing.

Out of all the positive experiences, the enhancement of children’s technological skills was the most reported by parents. Before COVID-19, the education system in some high-income countries gradually incorporated electronic devices to overcome barriers to education, such as distance and high absenteeism [25,26]. However, when the pandemic began, a rapid transformation occurred, and children had to digitalize their education. The literature argues that children’s technological skills are crucial not only for education but also for their future careers [26]. Multiple studies examined the role technology plays in advancing education. Digital transformation is inevitable in the modern world. Therefore, equipping children with the necessary technological skills is important, and countries that lack resources for web-based learning will face challenges [27,28].

Parents who had more positive experiences reported stress reduction after 1 year of web-based learning. The findings of this study confirmed that parents who adapted to web-based learning, used adaptive strategies, and viewed web-based learning as a positive event tended to experience less stress. Adaptive strategies such as planning, support, and proper communication with schools are important to facilitate the learning process, reduce stress, and enhance psychological health [29,30].

Participants with negative experiences reported greater obstacles in the education process, specifically having multiple learners within the same household. For web-based learning to be successful, proper collaboration between parents and schools and parental supervision during school days are needed. Parental engagement and supervision were reported by 81.7% of parents [24]. Parent involvement in the learning process requires parents to be attentive to children’s educational needs, to ensure an environment conductive to learning, and to play the role of the teacher. All these factors can be stressful and difficult for parents with multiple children undergoing web-based learning at the same time within the same household [31]. Obstacles to web-based learning can be culturally specific as well. An Arab household can contain multiple children with ages proximate to each other, which might not be a challenge for parents from a different culture. Parents with multiple children need to give their full attention and engagement to each child, which can be challenging for the parent and for the child [32].

### Limitations

Study data were collected from parents residing in the city of Jeddah, Saudi Arabia. Therefore, the findings might not be generalizable to parents in different cities and regions within the KSA. Another limitation is the gender of the parents. The number of mothers who participated was 3 times more than that of fathers. Therefore, the results of the study are less applicable to fathers.

The use of self-reporting limited the findings because self-reports can induce social desirability bias. This can be overcome through the use of more vigorous and objective measures. However, because of financial and time constraints, only self-reports were used. Another limitation was the study’s cross-sectional design. Cross-sectional study designs create ambiguity about the direction of the causal relationship between parental experiences and stress reduction. For example, did parents with positive experiences of web-based learning have lower stress, or was it the other way around?

### Conclusion and Recommendations

The findings of this research highlight the experiences parents underwent when their children studied via web-based learning during the COVID-19 pandemic. Parents’ experiences were more negative than positive, and parents with negative experiences reported more obstacles than parents with positive experiences. Regarding stress reduction after a year of web-based learning, parents with positive experiences were able to reduce their stress and adapt to their child’s web-based learning. Given the results of the study and the possible continuation of web-based learning even after the end of the pandemic, it is important that future research investigate the ramifications of web-based learning for children’s educational level, parents’ ability to manage children’s educational needs, and the support needed to be delivered to parents and children.

## Abbreviations

IRB: institutional review board
KSA: Kingdom of Saudi Arabia
